# Gain-of-function mutation in IFIH1 can cause both aicardi-goutières syndrome and systemic lupus erythematosus with IgA-deficiency

**DOI:** 10.1186/1546-0096-12-S1-P309

**Published:** 2014-09-17

**Authors:** Lien Van Eyck, Lien De Somer, Diana Pombal, Simon Bornschein, Francis de Zegher, Adrian Liston, Carine Wouters

**Affiliations:** 1 Microbiology and Immunology, KU Leuven, Belgium; 2 Autoimmune Genetics Laboratory, VIB, Belgium; 3 Pediatrics, UZ Leuven, Belgium; 4 Development and regeneration, KU Leuven, Leuven, Belgium

## Introduction

Gain-of-function mutations in *IFIH1* were identified in Aicardi-Goutières syndrome (AGS), a rare neuroimmunological disorder associated with elevated levels of type I interferon and characterized by leucoencephalopathy, brain atrophy and intracranial calcifications leading to profound intellectual disability, spasticity and dystonia. IFIH1 functions as an intracellular innate immune receptor that senses viral nucleic acids and leads to the induction of type I interferon and proinflammatory cytokines.

## Objectives

We aimed to identify the underlying genetic defect in a 16-year-old girl with severe early-onset and refractory systemic lupus erythematosus (SLE), IgA-deficiency and mild lower limb spasticity without neuroradiological manifestations.

## Methods

Whole-exome sequencing was performed on the index patient and her parents. Extensive immunological analysis was performed on serum and on peripheral blood mononuclear cells (PBMC) of the patient.

## Results

We independently identified the same *de novo* p.Arg779His *IFIH1* mutation in a young patient with severe refractory SLE with antiphospholipid syndrome as was described in two patients with an AGS phenotype without major immunological manifestations. The index patient in our study, a girl of European Belgian ancestry, was initially seen before the age of 1 year with frequent respiratory infections. Immunological testing revealed IgA-deficiency. By the age of 2.5 years she developed spasticity of the lower limbs, with normal cognitive and clinical development. Magnetic resonance imaging (MRI) of the brain and spine showed no abnormalities. At the age of 8 years the patient was diagnosed with SLE with secondary antiphospholipid syndrome. Clinically, she manifested arthritis, livedo rash, necrotizing cutaneous vasculitis and she developed a deep venous thrombosis. Blood analysis showed a marked inflammatory response, complement activation and highly increased levels of anti-dsDNA, anticardiolipin and anti-thyroid antibodies. Further analysis also showed elevated serum levels of interferon α and upregulation of IFIH1 on the RNA-level in patient PBMC. Single-photon emission computed tomography (SPECT) of the brain showed no signs of intracerebral vasculitis and brain CT and MRI did not show any microcalcifications or other abnormalities. Despite systemic immunosuppressive treatment, signs of autoimmune activation remained with persistently increased levels of circulating autoantibodies and complement activation, as well as persistently elevated interferon α in serum and IFIH1 upregulation in patient PBMC. Attempts at decreasing immunosuppressive medication were associated with disease flares. The lower limb spasticity has stabilized, cognitive functioning has remained excellent and all neuroradiological examinations have remained normal.

## Conclusion

This finding adds a new gene association to Mendelian lupus and greatly extends the disease spectrum associated with mutations in *IFIH1*, ranging from AGS to SLE with or without neuroradiological manifestations. This marked phenotypic heterogeneity, despite an identical mutation, demonstrates the importance of modifying genetic or environmental factors in type I interferon-dependent pathologies associated with mutations in *IFIH1*.

## Disclosure of interest

None declared.

